# Predictors for poor daily weight gain in preterm neonates exposed to different dose regimens of caffeine in ICU- a retrospective cohort study

**DOI:** 10.1186/s12887-024-04850-8

**Published:** 2024-06-20

**Authors:** Saeed Ahmed, Ayaz ur Rehman, Zainab Bibi, Sundus Iftikhar, Maleeha Raza, Hafiz Mohammad Amir Yousuf, Fizzah Naz, Syed Adil Mir Shah, Syeda Lamiya Mir, Ayesha Bibi, Wasif Ahmed Khan, Muhammad Sohail Salat, Gul Ambreen, Kashif Hussain

**Affiliations:** 1 Department of Pediatrics & Child Health, Aga Khan University Hospital, Karachi, Pakistan; 2 Dow University of Health Sciences, Karachi, Pakistan; 3 Department of Pharmacy , Aga Khan University Hospital, Karachi, Pakistan

**Keywords:** Caffeine citrate, Neonates, Apnea of prematurity, Side effects

## Abstract

**Background:**

With a wide therapeutic index, efficacy, ease of use, and other neuroprotective and respiratory benefits, caffeine citrate(CC) is currently the drug of choice for preterm neonates (PTNs). Caffeine-induced excessive energy expenditure, diuresis, natriuresis, and other CC-associated potential side-effects (CC-APSEs) result in lower daily-weight gain (WG) in premature neonates. This study aimed to evaluate the risk factors for daily-WG in neonates exposed to different dose regimens of caffeine in ICU.

**Method:**

This retrospective cohort study included neonates of ≤ 36weeks gestational age (GA) and received CC-therapy. The same participants were followed for data analysis in two postnatal phases: 15–28 and 29–42 days of life (DOL). Based on daily CC-dose, formed group-I (received; standard-doses = 5 mg/kg/day), group-II (received;>5-7 mg/kg/day), and group-III (received;>7 mg/kg/day). Prenatal and postnatal clinical characteristics, CC-regimen, daily-WG, CC-APSEs, and concomitant risk-factors, including daily-caloric intake, Parenteral-Nutrition duration, steroids, diuretics, and ibuprofen exposure, were analyzed separately for group-II and group-III using group-I as standard. Regression analysis was performed to evaluate the risk factors for daily-WG.

**Results:**

Included 314 PTNs. During 15–28 DOL, the mean-daily-WG(MD-WG) was significantly higher in group-I than group-II [19.9 ± 0.70 g/kg/d vs. 17.7 ± 0.52 *p* = 0.036] and group-III [19.9 ± 0.70 g/kg/d vs. 16.8 ± 0.73 *p* < 0.001]. During 29–42 DOL the MD-WG of group-I was only significantly higher than group-III [21.7 ± 0.44 g/kg/d vs. 18.3 ± 0.41 g/kg/d *p* = 0.003] and comparable with group-II. During 15–28 DOL, observed CC-APSEs was significantly higher in group-II and III but during 29–42 DOL it was only significant in group-III. In the adjusted regression analysis for daily-WG during 15-28DOL, with respect to standard-dose, 5-7 mg/kg/day (β=-1.04; 95%CI:-1.62,-0.93) and > 7-10 mg/kg/day (β=-1.36; 95%CI:-1.56,-1.02) were associated with a lower daily-WG. However, during 29-42DOL, this association was present only for > 7-10 mg/kg/day (β=-1.54; 95%CI:-1.66,-1.42). The GA ≤ 27weeks (β=-1.03 95%CI:-1.24, -0.88) was associated with lower daily-WG only during 15-28DOL. During both periods of therapy, higher cumulative-caffeine dose and presence of culture proven sepsis, tachypnea, hyponatremia, and feeding intolerance were significantly associated with lower daily-WG. Conversely, daily kcal intake was found to be linked with an increase in daily-WG in both periods.

**Conclusion:**

In this study cohort exposure to higher caffeine daily and cumulative doses is associated with lower postnatal daily-WG in PTNs than standard-daily doses, which may be due to its catabolic effects and CC-APSEs.

**Supplementary Information:**

The online version contains supplementary material available at 10.1186/s12887-024-04850-8.

## Background

Preterm neonates have immature respiratory control mechanisms and more than 85% of neonates born at gestational age (GA) ≤ 34 weeks, experience a developmental disorder known as apnea of prematurity (AOP) [1–3]. Preterm neonates have a higher probability of developing retinopathy of prematurity (ROP) and neurodevelopmental consequences due to apnea-associated intermittent hypoxemia [4, 5]. Likewise, AOP and poor respiratory drive increase the risk of extubation failure and prolonged mechanical ventilation in newborns experiencing respiratory distress [6].

Caffeine citrate (CC) is now recognized as a standard for treating AOP due to a better therapeutic index, comparable enteral bioavailability, and longer half-life [7–9]. Its use in preterm neonates is related to shorter mechanical ventilation dependence and lower chances of extubation failure [7]. Neonatal studies reported a reduced risk of bronchopulmonary dysplasia and an improved rate of very low birth weight (LBW) infants’ survival with no neurological disabilities at 18–21 months [10]. Apnea of prematurity might persist beyond 37 weeks postmenstrual age (PMA) in preterm neonates born at GA < 28 weeks [11]. Hence, CC is often prescribed for preterm neonates until they reach a PMA of 35–37 weeks in doses of 5–10 mg/kg in 24 h [3, 5, 7, 12, 13]. In this vulnerable population, the longer duration of CC-therapy and higher cumulative doses with potential catabolic response might affect the initial weight gain (WG) [14].

Previous studies reported a strong association between fetal growth restriction and high-dose maternal caffeine intake [15]. It is suggested that this phenomenon was consistent across all trimesters [15, 16]. A study reported that high-dose maternal caffeine intake resulted in LBW in 7% of neonates and small-for-gestational-age in 10%. Though, weight or head circumference reduction was not reported at the 18–21-month follow-up of participants subjected to CC-therapy for AOP. However, caffeine duration and dose-specific somatic effects through the neonatal phase were not determined [17]. Caffeine acts as a CNS stimulant by readily crossing the blood-brain barrier. It is an adenosine-G protein-coupled receptors antagonist [18, 19] and competitively inhibits the cAMP-phosphodiesterase enzyme, [Enzyme which converts cyclic-AMP to its noncyclic-form] therefore letting cAMP build up at the cellular level. Cyclic-AMP activates protein kinase-A to initiate the phosphorylation of glucose synthesizing certain enzymes. Thus, caffeine extends and intensifies the effects of adrenaline and adrenaline-like medications by blocking its removal [18, 20]. Consequently, caffeine intake results in increased heart rate, oxygen consumption, and metabolic rate [21]. Energy expenditure is increased by methylxanthines independent of physical activities and therefore enhances carbohydrate consumption in exposed neonates [16]. Compromised splanchnic blood flow is reported in the caffeine-exposed neonatal gut [22]. Caffeine intake is reported to result in diuresis and natriuresis [23–25]. A study reported a doubling of urine output with caffeine use in respiratory distress premature animal models [26]. The cumulative catabolic effect of caffeine exposure in preterm neonates through the above-mentioned mechanisms of action contributes to negatively influencing WG.

CC-serum half-life varies from 40 to 230 h, reducing with developing PMA up until 60 WOL. From the oral route, the peak CC-serum concentration is attained in 30–120 min and almost 85% of CC is excreted unchanged through the kidneys [27]. In preterm neonates, CC-metabolizing hepatic enzymes system matures with progressing gestation thus CC-clearance is markedly lesser with a greater volume of distribution [28, 29]. Consequently, this population is at greater risk of enduring adverse renal effects including natriuresis and diuresis [30, 31].

Caffeine is metabolized into three dimethylxanthines, (1) paraxanthine, (2) theobromine, and (3) theophylline [18]. Each of them exerts specific pharmacological effects. Paraxanthine boosts lipolysis and releases fatty acids and glycerol into the bloodstream to be consumed as fuel at the muscular level. Theobromine acts as a vasodilator resulting in increased nutrient flow to muscles and the brain. Theophylline has inotrope and chronotrope activities and thus increases contractility and heart rate [20]. Thus, the current clinical practices of prescribing caffeine in higher doses for a prolonged duration for AOP have a high potential to influence short-term neonatal growth, through its pharmacological effects of increasing metabolic rate, diuresis, risk of hyperglycemia, and catabolic tendency [15, 17, 32]. Other, side effects (SEs) involve tachycardia, tachypnea, hypertension, and increased sodium and calcium excretion [30, 33–35]. Necrotizing enterocolitis (NEC) is also a reported side effect of caffeine use but it has a strong association with several risk factors other than prematurity and the use of caffeine [36]. It is reported that the onset of adverse effects normally takes place at higher doses due to higher serum concentrations [37, 38]. Few recent studies have reported an association with a higher risk of the onset of osteopenia in preterm neonates exposed to higher CC-dose [39, 40].

Consequently, this retrospective cohort study aimed to evaluate the potential risk factors for MD-WG in preterm neonates exposed to three different intravenous or oral dosage regimens of CC-therapy for managing AOP and obtaining other respiratory and neurological benefits in preterm neonates (5, > 5–7, and > 7–10 mg/kg/day) [41].

## Methods

### Study design, setting, and population

This study was performed in Aga Khan University Hospital (AKUH), a tertiary care setting in Karachi, Pakistan. The AKUH has the facility of 24 bedded multispecialty tertiary care NICU. About 1200 neonates are admitted annually with the influx of very preterm high-risk newborns from all over the country. All the preterm neonates (GA ≤ 36 weeks) were included in the initial cohort who were admitted to NICU and were administered intravenous or oral CC-therapy for managing AOP and obtaining other respiratory and neurological benefits in preterm neonates during the study period (April 2017-December 2018). Neonates were identified from the hospital’s electronic database. All the neonates with fluid restrictions, congenital anomalies, and ≥ grade-III intraventricular hemorrhage (IVH) were excluded. Neonates with NEC (modified Bell’s stage-2b and beyond) were also excluded due to their strong association with several risk factors other than prematurity and the use of caffeine [36]. Additional exclusion criteria were the concurrent prolonged exposure to other WG-influencing medications, such as diuretics, corticosteroids, and ibuprofen. Neonates who died before entering phase-II of the study were also excluded.

### Unit protocol for use of caffeine

Following the unit protocol, intravenous (IV)-CC was administered initially and was switched to the oral-CC subsequently when tolerating adequate enteral daily feed of > 100 ml/kg/day. We included all the neonates who received CC-therapy, either IV or oral, as the bioavailability of CC is almost similar through oral and IV routes [42, 43]. Maternal caffeine/tobacco exposure was not an exclusion criterion, and no information was retrieved in this regard. Likewise, neonatal route of nutrition intake [enteral nutrition (EN) vs. parenteral nutrition (PN)], type of enteral feed (mother milk vs. formula feed), or caloric/protein concentration in formula feed were not considered as exclusion criteria. During the study period, Enfamil® Premature 20 formula feed was used in the unit, where mother/donor milk was not available.

### Data collection

Data were extracted from institutional electronic health records, including antenatal, prenatal, and postnatal clinical characteristics such as maternal parity level, inter-delivery interval, gender, GA, birth weight, route and type of nutrition. Details about the CC-regimen included route and duration of therapy, loading (mg/kg), daily (mg/kg/day), cumulative doses (mg/kg), and the number of neonates who needed dose reduction or withheld of CC-therapy due to SEs. In addition, data were retrieved about the other concomitant risk factors affecting neonatal WG including exposure to steroids, diuretics, and ibuprofen. Nutritional data included the type and amount of EN and duration of PN. Duration of PN counts for total PN and partial PN days, as in the study center PN is continued till > 100 ml/kg/day EN tolerance [39]. Other study variables were obtained from serially recorded weight and clinical progress charts from the nursing notes for CC-APSEs. Laboratory parameters included serum sodium (Na), potassium, and glucose levels.

### Intervention and outcome measurement

Neonates during the study period received licensed neonatal products (Peyona, CC 20 mg/mL solution by Chiesi Ltd.) for IV and oral use. An intravenous loading dose of 20 mg/kg was followed by a once-daily maintenance dose of 5-10 mg/kg CC (equivalent to 2.5-5 mg/kg caffeine base) 24 h after the loading dose according to unit protocol. Intravenous-CC was switched to the oral route at the achievement of sufficient (> 100 ml/kg/day) EN [10, 17]. . The daily dose and duration of CC-therapy were entirely decided by the teams responsible for neonatal care.

For the current study, MD-WG in g/kg/day was assessed for included neonates during two specified periods: (i) 15–28 days of life (DOL) and (ii) 29–42 DOL. The outcomes were analysed in two phases because caffeine therapy is continued in preterm neonates for longer duration. Importantly, like many other physiological and metabolic processes in preterm neonates, pharmacokinetics also exhibits a relative immaturity that changes postnatally. The lack of activity of metabolizing enzymes can be responsible for extreme toxicity syndromes. Maturation of drug metabolizing enzymes is therefore an important factor in determining drug dose selection in preterm neonates [44].

In this study, we used the MD-WG record from the fifteenth DOL onwards, as up to 14% mean physiological body weight loss is reported in preterm neonates in the first week of life (WOL) and then starts to regain during the second WOL. Based on daily CC-dose/kg of 14th DOL and 28th DOL all the included neonates were further divided into three groups (Gp) for phase-I and phase-II respectively.

Gp-I included all the neonates who received 5 mg/kg/day.

Gp-II included neonates who were exposed to > 5-7 mg/kg/day.

Gp-III had neonates who were exposed to > 7-10 mg/kg/day.

According to clinical practices, there was the possibility of switching neonates from one group to another due to an increment in birth weight with the advancement of age or dose reduction due to side effects or dose increment to achieve the desired clinical outcomes. During the same phase neonates facing these scenarios were kept in the initially assigned group but while making groups for phase-II we considered the daily dose of 28th DOL. Therefore, we retrieved data for these SEs, the number of neonates that needed dose adjustments, and the cumulative caffeine dose in each phase. Following the unit practice, experienced neonatal nursing staff measured the neonatal weight from birth to discharge daily and record the changes. Less frequent weight measures were done if clinically suggested [45]. . Other CC-APSEs were also observed in all groups. Measurement of serum CC-concentrations was not performed in this study [46].

### Statistical analysis

Data were analyzed using STATA version 17.0. To evaluate the effects of different CC daily dose regimens, Gp-II and Gp-III were separately compared with the standard dose group. Descriptive statistics were computed for categorical variables and presented as frequencies with percentages. A Chi-square test was performed for comparing the percentage differences between the study groups. Quantitative variables were presented as mean ± SD and median (IQR). T-test was performed for comparison of two group means, whereas the Wilcoxon rank-sum test was performed for non-parametric testing. A p-value < 0.05 was considered statistically significant.

In this study, we applied linear regression analysis to determine the association between the independent factors and the relevant dependent variable. we used linear regression analysis to evaluate the individual effects of each predictor variable on the response variable. Univariate analysis was first performed, a more liberal threshold of *p* < 0.20 was used as the criterion for inclusion in the model. Multivariate linear regression analysis was then carried out to investigate how the chosen variables together affected the dependent variable. The multivariate model was used to evaluate the unique contributions of the variables that satisfied the first *p* < 0.20 criterion in the univariate analysis while taking additional predictors into consideration. The goal of this stage was to improve the model and find the most reliable predictors that could account for variations in the dependent variable on their own.

### Ethical approval and consent to participate

Before performing this study, ethical approval was taken from the institutional ethical committee of Aga Khan University Hospital (ERC # 2019-2111-5600) and informed consent from a parent or guardian for participants was waived by ERC as all the data was collected retrospectively.

## Results

During the study period, a total of 527 preterm neonates received caffeine. Applying exclusion criteria, a total of 243 neonates were excluded and finally, 314 neonates were included in the study (Supplementary File- Fig. 1). Based on daily CC dose/kg of 14th DOL, during phase-I 129 neonates received caffeine 5 mg/kg/day (Gp-I), 128 neonates received > 5-7 mg/kg/day (Gp-II), and 57 neonates were on > 7-10 mg/kg/day (Gp-III). The same 314 participants were followed in the second phase. In this phase based on the daily CC-dose/kg of 28th DOL 201 neonates fell in Gp-I, 81 in group-II, and 32 neonates made Gp-III. Gp-II and Gp-III were separately compared with Gp-I. The demographic and clinical characteristics of all study participants are comparable and statistically, no significant differences were found (Table 1).

**Table 1:** Baseline demographic and clinical characteristics of preterm neonates who received caffeine therapy

	Study groups based on caffeine daily dose			*p*-value	
Dose of CC, mg/kg/day	Group-I *N* = 129	Group-II *N* = 128	Group-III *N* = 57	Group-I vs. Group-II	Group-I vs. Group-III
**Gender**					
Male	75 (58.1%)	78 (60.9%)	35 (27.3%)	0.46	0.062
**Birth weight (gm)**					
Mean ± SD	1154.4 ± 292.8	1133.8 ± 373.2	1137.7 ± 300.6	0.69	0.75
**BW (gm)**					
≤1000	48 (37.2%)	56 (43.8%)	23 (40.4%)	0.17	0.60
1001–1500	78 (60.5%)	64 (50.0%)	32 (56.1%)		
1501–2500	3 (2.3%)	8 (6.3%)	2 (3.5%)		
**Gestational age (weeks)**					
Mean ± SD	29.0 ± 1.5	29.3 ± 2.0	28.6 ± 2.1	0.31	0.14
**GA (weeks)**					
≤27	16 (12.4%)	17 (13.3%)	8 (14.0%)	0.35	0.47
>27-≤31	109 (84.5%)	97(75.8%)	45 (78.9%)		
>31-≤36	4 (3.1%)	14 (10.9%)	4 (7.0%)		
**GDM mother**					
Yes	26 (20.2%)	24 (18.8%)	13 (22.8%)	0.72	0.86
**Maternal parity level**					
Low < 2	58(44.9%)	84(65.6%)	31 (54.4%)	0.096	0.38
Moderate 2–4	51(39.5%)	28 (21.9%)	19 (33.3%)		
High > 4	20 (15.5%)	16 (12.5%)	7 (12.2%)		
**Inter-Delivery Interval**					
6–17 months	13 (10.1%)	15 (11.7%)	6 (10.5%)	0.82	0.91
18–36 months	48 (37.2%)	38 (29.7%)	18 (31.6%)	0.77	0.62
37–60 months	15 (11.6%)	14 (10.9%)	4 (7.0%)	0.85	0.51
61–180 months	5 (3.9%)	7 (5.5%)	4 (7.0%)	0.62	0.28
**Primary gravida**	48 (37.2%)	54 (42.2%)	25 (43.9%)	0.30	0.33
**Pregnancy-induced hypertension/pre-eclampsia**					
Yes	37 (28.7%)	28 (21.8%)	15 (26.3%)	0.25	0.68
**Antenatal steroids (2 doses)**					
Yes	52 (40.3%)	55 (42.9%)	22 (38.6%)	0.81	0.73
**Emergency lower segment cesarean section**					
Yes	78 (60.6%)	65 (50.8%)	27 (47.4%)	0.11	0.098
**5-Minute Apgar score of < 5**					
Yes	35 (27.1%)	32 (25.0%)	13 (22.8%)	0.50	0.27
Age at intubation (h) *					
Median (IQR)	5 (3–9)	4 (2–9)	4 (2–10)	0.73	0.48
**Duration of intubation (days)**					
Median (IQR)	12 (7–25)	10 (5–23)	10 (7–24)	0.46	0.70
**Route of Nutrition**					
Received Parenteral Nutrition	111 (86.0%)	117 (91.4%)	50 (87.7%)	0.86	0.99
**Duration of Total Parenteral Nutrition (days)**					
Median (IQR)	8 (5–21)	7 (5–19)	9 (7–25)	0.47	0.16
**Type of Nutrition**					
Received Breast Milk	73 (56.6%)	78 (60.9%)	31 (54.4%)	0.53	0.81
Received Formula milk ^@^	56 (43.4%)	50 (39.1%)	26 (45.6%)	0.96	0.47
**kcal/day**					
Mean ± SD	132.2 ± 6.7	131.7 ± 7.4	133.4 ± 6.8	0.84	0.17
**Duration of intravenous caffeine (days)**					
Mean ± SD	16.7 ± 10.6	15.2 ± 12.5	19.6 ± 7.5	0.82	0.29

Group-I received ≤ 5 mg/kg/day; Group-II received > 5-7 mg/kg/day; Group-III received > 7-10 mg/kg/day

Note: Gp-I was separately compared with Gp-II and Gp-III

^@^Nestle Pre NAN® formula

The maximum daily and cumulative CC-doses received by Gp-II and Gp-III during 15–28 DOL and 29–42 DOL were compared with Gp-I and shown in Table 2. During phase-I a significantly higher MD-WG was observed in group-I compared with group II [19.9 ± 0.70 g/kg/d vs. 17.7 ± 0.52 *p* = 0.036] and Gp-III [19.9 ± 0.70 g/kg/d vs. 16.8 ± 0.73 *p* < 0.001] (Supplementary File- Fig. 2). The MD-WG pattern was also similar in phase-II and a higher increment was observed in Gp-I when compared to Gp-II and Gp-III. Statistically, this difference was not significant in this period when comparing Gp-I with Gp-II [21.7 ± 0.44 g/kg/d vs. 19.4 ± 0.42 g/kg/d *p* = 0.243]. However, the difference between the MD-WG of Gp-I was significantly higher than Gp-III [21.7 ± 0.44 g/kg/d vs. 18.3 ± 0.41 g/kg/d *p* = 0.003] (Table 2 and Supplementary File-Fig. 3).

**Table 2:** Study outcomes of Premature Infants Exposed to Caffeine Citrate at Three Different Doses During Two Distinct Time Frames

Day of life (DOL)	15–28 days			*p*-value		29–42 days			*p*-value	
	Group-I *N* = 129	Group-II *N* = 128	Group-III*N* = 57	Group-I vs. Group-II	Group-I vs. Group-III	Group-I *N* = 201	Group-II *N* = 81	Group-III *N* = 32	Group-I vs. Group-II	Group-I vs. Group-III
**Caffeine Regimen and Mean Daily Weight Gain**										
Maximum daily dose of caffeine (mg/kg/day)	5.8 ± 1.2	6.5 ± 1.5	8.9 ± 2.3	< 0.001	< 0.001	5.4 ± 1.0	7.2 ± 2.1	8.9 ± 1.9	< 0.001	< 0.001
Cumulative dose of caffeine (mg/kg)	65.3 ± 8.5	85.4 ± 9.2	110.6 ± 6.9	< 0.001	< 0.001	59.4 ± 8.9	87.0 ± 7.5	104.3 ± 5.7	< 0.001	< 0.001
Mean weight gain (g/kg/d)	19.9 ± 0.70	17.7 ± 0.52	16.8 ± 0.73	0.036	< 0.001	21.7 ± 0.44	19.4 ± 0.42	18.3 ± 0.41	0.243	0.003
**Frequency of caffeine-associated potential side effects**										
**Tachycardia**	42 (32.6%)	51 (39.8%)	23 (40.4%)	**0.042**	**0.014**	35 (17.4%)	17 (20.9%)	12 (37.5%)	0.83	**0.043**
**Hypertension**	25 (19.4%)	20 (15.6%)	12 (21.1%)	0.63	0.51	12 (5.9%)	6 (7.4%)	4 (12.5%)	0.31	0.23
**Tachypnea**	36 (27.9%)	42 (32.8%)	25 (43.9%)	0.29	**0.003**	20 (9.9%)	11 (13.5%)	9 (28.1%)	0.23	**0.005**
**Hypokalaemia**	13 (10.1%)	19 (14.8%)	16 (28.1%)	0.20	**0.003**	14 (6.9%)	6 (7.41%)	6 (18.8%)	0.87	0.11
**Hyponatremia**	45 (34.9%)	57 (44.3%)	26 (45.6%)	**0.045**	**0.021**	35 (17.4%)	21 (25.9%)	21 (65.6%)	0.075	**0.011**
**Polyuria**	41 (31.8%)	48 (37.5%)	30 (52.6%)	0.71	**0.003**	13 (6.5%)	8 (9.8%)	9 (28.1%)	0.26	**0.009**
**Tremors**	10 (7.8%)	15 (11.7%)	5 (8.8%)	0.31	0.59	8 (3.9%)	3 (3.7%)	1 (3.1%)	0.82	0.62
**Vomiting**	22 (17.1%)	24 (18.8%)	13 (22.8%)	0.69	0.17	13 (6.5%)	8 (9.8%)	3 (9.3%)	0.23	0.28
**Feed intolerance**	21 (16.3%)	22 (17.2%)	19 (33.3%)	0.68	**0.003**	12 (5.9%)	9 (11.1%)	15 (46.8%)	0.35	**< 0.001**
**Hyperglycaemia**	7 (5.4%)	16 (12.5%)	8 (14.0%)	**0.021**	**0.044**	4 (2.0%)	3 (3.7%)	2 (6.3%)	0.43	0.49

Group-I received ≤ 5 mg/kg/day; Group-II received > 5-7 mg/kg/day; Group-III received > 7-10 mg/kg/day. Data presented as mean ± SD. Note: Gp-I was separately compared with Gp-II and Gp-III

During both study periods (15–28 DOL and 29–42 DOL), the frequency of CC-APSEs in neonates exposed to the standard dose group (Gp-I) was separately compared with neonates of Gp-II and Gp-III (Table 2). During 15–28 DOL tachycardia, hyponatremia, and hyperglycemia were observed in a significantly higher number of neonates in Gp-II and Gp-III in comparison with Gp-I. Though, during the same period tachypnea, hypokalemia, polyuria, and feed intolerance were significantly higher only in Gp-III when compared with Gp-I. However, other SEs including vomiting, tremors, and hypertension were found without significant difference (Table 2). During 29–42 DOL, CC-APSEs were comparable between Gp-II and Gp-I. However, a significantly higher number of neonates in Gp-III reported tachycardia (*p* = 0.043), tachypnea (*p* = 0.005), hyponatremia (*p* = 0.011), polyuria (0.009), and feed intolerance (p = < 0.001) (Table 2). In phase-I and II no dose reduction was required in Gp-I. However, 25 (19.5%) and 8 (9.9%) neonates in Gp-II and 14 (24.5%) and 13 (40.6%) neonates in Gp-III needed dose reduction due to CC-APSEs in phase-I and II, respectively. Not a single participant’s daily caffeine dose was withheld in any phase.

In the adjusted regression analysis for weight gain between 15 and 28 days, certain factors were identified as influencing the mean weight gain. Notably, female gender was associated with a decrease in mean weight gain (β=-0.45; 95% CI: -0.87, -0.03), along with GA ≤ 27weeks (β =-1.03 95% CI: -1.24 -0.88), parenteral route of nutrition, presence of culture proven sepsis and complications such as tachypnea, hyponatremia, polyuria, hyperglycemia and feeding intolerance. Conversely, daily kcal intake was found to be linked with an increase in mean weight gain (β = 1.24; 95% CI: 0.44, 2.05). With respect to standard dose, 5-7 mg/kg/day (β=-1.04; 95% CI: -1.62, -0.93) and > 7-10 mg/kg/day (β=-1.36; 95% CI: -1.56, -1.02) doses were associated with a decrease in mean weight gain. Cumulative caffeine (β=-1.12; 95% CI: -1.28, -0.75) dose was also identified as inverse weight influencing factors (Table 3).

**Table 3:** Associated risk factors for daily weight gain in preterm neonates

	Mean weight gain (15–28 days)				Mean weight gain (29–42 days)			
	Unadjusted		Adjusted		Unadjusted		Adjusted	
	β Coefficient (95% CI)	*P*-value	β Coefficient (95% CI)	*P*-value	β Coefficient (95% CI)	*P*-value	β Coefficient (95% CI)	*P*-value
**Caffeine daily dose (mg/kg/day)**								
≤ 5	Ref.				Ref.		Ref.	
5–7	-1.06 (-1.78, -0.86)	< 0.001	-1.04 (-1.62, -0.93)	0.021	-1.01 (-1.56, -0.58)	0.002	0.67 (-0.92, 1.25)	0.405
> 7–10	-1.59 (-1.72, -1.12)	< 0.001	-1.36 (-1.56, -1.02)	< 0.001	-1.54 (-1.56, -1.31)	0.002	-1.54 (-1.66, -1.42)	< 0.001
**Cumulative dose of caffeine (mg/kg)**	-1.16 (-1.38, -0.80)	< 0.001	-1.12 (-1.28, -0.75)	0.027	-1.15 (-1.44, -0.62)	0.001	-1.12 (-1.72, -1.10)	< 0.001
**Gender**								
Male	Ref.		Ref.		Ref.			
Female	-0.43 (-0.91, 0.05)	0.076	-0.45 (-0.87, -0.03)	0.037	-0.29 (-0.81, 0.23)	0.267		
**BW (gm)**								
≤1000	-1.03 (-1.46, -0.79)	0.003			-1.21 (-1.37, -0.82)	0.002		
1001–1500	0.22 (-1.24, 1.68)	0.767			-1.14 (-1.35, -0.93)	0.025		
1501–2500	Ref.				Ref.			
**GA (weeks)**								
≤27	-1.05 (-1.34, -0.72)	0.005	-1.03 (-1.24 -0.88)	0.024	-1.25 (-1.31, -0.92)	0.004		
>27-≤31	0.22 (-1.24, 1.68)	0.767			-1.14 (-1.35, -0.93)	0.025		
>31-≤36	Ref.				Ref.			
**LOS-NICU (days)**	-0.01 (-0.03, 0.01)	0.49			-0.01 (-0.04, 0.01)	0.23		
**LOS-Hospital(days)**	-0.01 (-0.03, 0.02)	0.592			-0.01 (-0.04, 0.01)	0.347		
**Culture Proven Sepsis**	-1.41 (-1.89, -0.96)	< 0.001	-0.97 (-1.47, -0.42)	< 0.001	-1.33 (-2.06, -1.01)	< 0.001	-1.31 (-1.95, -0.82)	< 0.001
**Number of siblings**	0.03 (-0.09, 0.14)	0.661			0.01 (-0.12, 0.14)	0.918		
**Multiple Births**	0.18 (-0.33, 0.21)	0.362			-0.05 (-0.77, 0.68)	0.904		
**Inter-Delivery Interval (Months)**	1.28 (0.55, 2.22)	0.004			1.50 (0.56, 2.84)	0.007		
**Antenatal steroids (2doses)**	-0.1 (-0.58, 0.38)	0.685			-0.33 (-0.85, 0.19)	0.217		
**Emergency lower segment caesarean section**	0.17 (-0.31, 0.64)	0.498			-0.05 (-0.57, 0.47)	0.84		
**5-Minute Apgar score of < 5**	0.26 (-0.3, 0.82)	0.362			0.05 (-0.57, 0.66)	0.88		
**Age at intubation (h)**	0 (-0.08, 0.08)	0.968			-0.02 (-0.1, 0.06)	0.563		
**Route of Nutrition -Received Parenteral Nutrition**	1.29 (0.37, 2.71)	0.003	1.31 (0.46, 2.19)	0.007	0.26 (-1.20, 1.88)	0.834		
**Type Of Nutrition**								
Received Breast Milk	Ref.				Ref.			
Received Formula milk ^@^	-1.13 (-1.25, -0.90)	0.020			0.26 (-1.22, 1.59)	0.731		
**Duration of PN (days)**	-0.02 (-0.07, 0.03)	0.373			-0.01 (-0.06, 0.04)	0.576		
**kcal/day**	1.45 (0.56, 2.35)	0.002	1.24 (0.44, 2.05)	0.003	1.54 (0.56, 2.51)	0.002	1.54 (0.66, 2.42)	0.001
**Complications**								
Tachycardia	-1.4 (-1.88, -0.93)	< 0.001	-0.99 (-1.45, -0.53)	< 0.001	-1.54 (-2.04, -1.03)	< 0.001	-1.34 (-1.92, -0.75)	< 0.001
Hypertension	-1 (-1.61, -0.39)	< 0.001			-1.2 (-1.86, -0.54)	< 0.001		
Tachypnea	-1.34 (-1.82, -0.85)	< 0.001			-1.35 (-1.88, -0.82)	< 0.001		
Hypokalemia	-1.18 (-1.86, -0.5)	< 0.001			-1.6 (-2.32, -0.88)	< 0.001		
Hyponatremia	-1.09 (-1.56, -0.62)	< 0.001	-0.99 (-1.45, -0.53)	< 0.001	-0.99 (-1.5, -0.48)	< 0.001	-1.47 (-2.85, -0.09)	0.037
Polyuria	-0.79 (-1.27, -0.3)	< 0.001	-0.98 (-1.43, -0.55)	< 0.001	-0.64 (-1.18, -0.11)	0.018		
Tremors	-1.42 (-2.26, -0.58)	< 0.001			-1.55 (-2.45, -0.66)	0.001		
Vomiting	-1.26 (-1.86, -0.65)	< 0.001			-1.5 (-2.15, -0.85)	< 0.001		
feed intolerance	-1.96 (-2.53, -1.4)	< 0.001	-1.28 (-1.86, -0.71)	< 0.001	-1.93 (-2.53, -1.32)	< 0.001	-1.89 (-2.67, -1.11)	< 0.001
Hyperglycemia	-2.6 (-3.44, -1.76)	< 0.001	-0.99 (-1.45, -0.53)	< 0.001	-2.89 (-3.75, -2.03)	< 0.001		

BW = Birth weight; GA = gestational age; LOS = length of stay

In the adjusted analysis for weight gain between 29 and 42 days, with respect to standard dose, only > 7-10 mg/kg/day (β=-1.54; 95% CI: -1.66, -1.42) dose was associated with a decrease in mean weight gain. Cumulative caffeine dose (β=-1.12; 95% CI: -1.72, -1.10) was also identified as inverse weight influencing factors in this period. In this period the presence of culture proven sepsis, tachypnea, hyponatremia, and feeding intolerance had a strong association to reduce the daily weight gain. Conversely, daily kcal intake was found to be linked with an increase in daily weight gain (β = 1.54; 95% CI: 0.66, 2.42) (Table 3).

## Discussion

Our study identified higher daily and cumulative caffeine doses with respect to standard dose, as a significant influencing factor to reduce the daily neonatal weight gain during the 15–28 DOL. Although during 29–42 DOL only > 7-10 mg/kg/day and higher cumulative dose were associated to inversely effect the weight gain. In addition, tachycardia, hyponatremia, and feed intolerance were associated complications, which negatively influenced the weight gain during 15–28 DOL and 29–42 DOL. The major finding of this study is that the neonates exposed to 5–7 mg/kg/d initially had lower MD-WG but during 29–42 DOL their MD-WG was comparable with neonates exposed to a standard daily dose of 5 mg/kg/day. On the other hand, the MD-WG in neonates exposed to > 7 mg/kg/d was significantly lower till the sixth WOL compared to neonates exposed to standard doses.

Previous recent studies reported initial higher daily weight loss in caffeine-treated premature neonates through the first 21 DOL and then a gradual reduction in daily weight loss in subsequent DOL [10, 47]. However, in our study, the higher daily and cumulative CC-doses were linked with lower MD-WG till 6th WOL in neonates who received > 7 mg/kg/day. Similar results are reported in another recent study [38] but they compared only two doses regimens that are 5 and 10 mg/kg/d.

The recommended daily CC-dose ranges from 5 to 10 mg/kg/d, which gives clinicians a wide range to prescribe for LBW and VLBW neonates. This wide range, however, needs to be evaluated in terms of its useful and undesirable effects on these vulnerable populations. To the best of our knowledge, this is the first study that compared two different high dose caffeine regimens with standard daily doses regime in preterm neonates. And evaluated the associated risk factors for daily weight gain. Previous studies have shown similar respiratory advantages from 2.5 to 5 mg/kg/d of caffeine base and therefore throughout higher maintenance daily dose is debatable [10, 38, 47]. A prospective cohort study proposed that earlier initiation of CC-therapy reduces the incidence of IVH and invasive ventilation requirements [48]. In addition, a recent randomized trial (RT) preferred the use of twice-daily caffeine dosage [49]. Another trial suggested that a higher daily CC-dose regimen possibly decreases the risk of extubation failure [3]. Though, a more recent RT has not demonstrated earlier extubation from earlier CC-therapy initiation [50].

A European trial reported about twenty-one CC-APSEs in premature neonates [51]. The CC-APSEs of the current study are the same reported in previous studies, including tremors, vomiting, hyperglycemia, and hypokalemia [33]. Other, SEs involve tachycardia, tachypnea, hypertension [35] increased urinary output [30], sodium, and calcium excretion [34]. It is reported that the onset of CC-APSEs normally takes place at higher doses leading to higher serum concentration [37].

Romagnoli et al. [52] tested the frequency of CC-APSEs of two different dose regimens for preventing idiopathic AOP in premature neonates. They reported a greater number of neonates exposed to higher daily maintenance doses who experienced CC-APSEs including vomiting, feed intolerance, regurgitation hyperglycemia, and tachycardia. A higher daily CC-dose could promote a faster CC-metabolism into theophylline and other methylxanthine metabolites. These were transient manifestations and mostly sometimes reverted or resolved during CC-therapy in the following weeks. Our results are similar and even more specific in terms of daily dose. In our study, most of the SEs resolved in subsequent weeks in neonates exposed to ⩽ 7 mg/kg/day but neonates exposed to a higher dose of > 7 mg/kg/d experienced several SEs till the sixth WOL. This evolving response can be explained by the lack of activity of drug metabolizing enzymes in preterm neonates and their maturation and development in postnatal age [44].

Currently recommended daily CC-doses expose premature neonates to a significantly higher caffeine dose than at any other age. A daily maintenance CC-dose of 5–10 mg/kg is frequently given to neonates born at 25–32 weeks gestation and have a body weight of 500–1500 g. The CARE and other recent studies demonstrated that this caffeine amount is more than the amount received from dietary sources during the antenatal period [15, 53]. According to a recent systematic review that highlighted the dose–response relationship between caffeine exposure in pregnancy and suboptimal fetal WG. Results are suggestive of causation and do not indicate a threshold effect [54].

Caffeine has psychoactive properties thus influencing the sleep cycle and WG pattern through its somatic effects by reducing the overall time and worsening the quality of sleep [55]. Along with these effects and dose-dependent SEs and their intensity and duration can affect the overall clinical outcomes. These observations advocate the vigilant use of CC in premature neonates with a customized regimen. Our study aimed to analyze the clinical advantages of CC based on different dose regimens including length of NICU and hospital stays. Higher daily dose exposure not only restrained the WG but also worsened the clinical condition by exposing the neonates to undesirable side effects. Resulting in longer NICU and hospital stays.

Dose-dependent polyuria, hyponatremia, and feed intolerance in neonates exposed to higher daily doses are other major contributing factors for lower WG. A study established a dose-response relationship between CC-doses and electrolytes concentrations in serum and urine [56]. The author stated a substantially higher sodium (Na) excretion accompanying diuresis at higher caffeine doses. The probable causes of caffeine-induced natriuresis are caffeine A1-adenosine receptor antagonism and proximal tubular Na-reabsorption inhibition [57–59]. Secondly through caffeine A2-adenosine receptor antagonism and reduced distal Na-reabsorption [42, 60]. However, the underlying mechanisms are still unexplained. The mean MD-WG during the 15–28 DOL is higher than the 29–42 DOL. This might be related to the fact that most of the neonates received optimal nutrition through total parenteral nutrition (PN) or partial PN during 15–28 DOL. However, during the study duration, we did not have breast milk fortifier practices in our NICU.

Caffeine is a relatively safer drug to treat AOP in preterm neonates maybe make practitioners less cautious about individualizing the caffeine regimen thus prescribing unnecessary higher doses for a prolonged period resulting in possible SEs [37, 39]. We need to explore all recent respiratory support developments including nasal high-flow humidified O2-therapy or low-flow systems, continuous positive nasal pressure accommodated with synchronization/backup breaths, neonatal postural adjustment, and advancements in neonatal care procedures, to circumvent unnecessary dependence on CC-usage. Schmidt et al. reported that CC-therapy used for treating AOP was not related to significantly reducing the collective rate of motor, behavioral, and academic impairments nonetheless had an association to reduce the risk of motor impairment [61]. Recent literature supports the positive role of CC-therapy in managing AOP and might be associated to reduce incidences of chronic lung disease, however, may not be to the extent it is practiced [62].

Some inherent limitations were there in the present study because of its retrospective observational nature and not an RT to make it more conclusive. The selection of CC-dose for any neonate was the clinicians’ decision ranging from 5 to 10 mg/kg/d and there was a likelihood of treating sicker neonates with higher doses, who were not responding to the standard dose. There was no practice of testing serum CC-concentrations in our NICU. Although the MD-WG estimation was obtained from daily weight measurement, in a few neonates, thrice-weekly weight was measured due to some clinical limitation however, this was reflected across all neonatal groups. Though this retrospective study compared most of the factors influencing daily weight gain in neonates but data for maternal socioeconomic status could not be obtained.

Despite a few limitations, our study has several strengths. This is the first study that compared two different high dose caffeine regimens with standard daily CC-dose regimens in preterm neonates and evaluated the association of other risk factors for daily weight gain. During the study period all the eligible neonates were included in the study. Hence, with a large number of participants study was completed on the same neonates in 15–28 DOL and 29–42 DOL. During the study period, no major changes took place in the NICU practice guidelines. All the participants received similar feeding regimens. Days on PN and total caloric intake were comparable in all groups. Throughout the study period, the same caffeine pharmaceutical brand was used for intravenous and oral dosages for all participants. The first two WOL were excluded for all participants when the possibility of relatively physiological weight loss is greater. Neonates were observed from the third WOL onwards, which is the more stable and growing phase hence increasing more clinical significance and relevance to the results of our study.

## Conclusion

Exposure to higher daily and cumulative doses of caffeine in this study cohort is associated with lower postnatal weight gain in preterm neonates than standard daily doses may be due to its catabolic effects in higher doses. This study also concludes that neonates exposed to higher doses have more frequently experienced potentially undesirable SEs and they persist for several weeks, a risk factor for lower weight gain and longer hospital stay. Our results warrant a vigilant approach from neonatal practitioners for the use of higher caffeine doses in the preterm neonate. Further RCTs are needed for the validation of these outcomes and to establish the most effective and safest caffeine regimens for the treatment of apnea of prematurity in premature neonates.

### Electronic supplementary material

Below is the link to the electronic supplementary material.

Supplementary Material 1

## Data Availability

The datasets used and/or analyzed during the current study are available from the corresponding author upon reasonable request.

## Abbreviations

CC: Caffeine Citrate
AOP: Apnea of Prematurity
PTNs: Preterm Neonates
CC-ASPEs: CC-Associated Potential Side Effects
MD-WG: Mean Daily Weight Gain
GA: Gestational Age
WOL: Week of Life
DOL: Days of Life
PN: Parenteral Nutrition
ROP: Retinopathy of Prematurity
LBW: Low Birth Weight
PMA: Postmenstrual age
SEs: Side Effects
IVH: Intraventricular Hemorrhage
EN: Enteral Nutrition
RT: Randomized Trial
