# Supplementary material for: Predictors for poor daily weight gain in preterm neonates exposed to different dose regimens of caffeine in ICU- a retrospective cohort study
Source: BMC Pediatr. 2024 Jun 20;24:401. doi: 10.1186/s12887-024-04850-8 (PMC11188204; doi:10.1186/s12887-024-04850-8)
Supplement: Supplementary file 1 — Supplementary Material 1 [file 12887_2024_4850_MOESM1_ESM.docx]

**Figures**

**IDENTIFICATIO**N

Preterm neonates admitted to NICU with ≤ 36 weeks of GA and started prophylactic caffeine therapy for AOP.

n=527

**ELIGIBILITY**

213 Excluded

56 Neonates died before entering phase II.

107 Discharged/transferred before entering phase II.

3 congenital anomalies

12 ≥ grade-III intraventricular Hemorrhage (IVH)

6 fluid restrictions

16 diagnosed with necrotizing enterocolitis (NEC)

13 concurrently received other drugs that could influence neonatal weight gain

**INCLUSION**

314 Neonates

Included

**Groups based on Daily CC-dose of 14^th^ DOL (phase-I)**

**PERIOD-I (15-28 DOL)**

Gp-I (Standard dose Gp) n= 129

Gp-III (>7-10mg/kg/day) n= 57

Gp-II (>5-7mg/kg/day) n= 128

**Groups based on Daily CC-dose of 28^th^ DOL (phase-II)**

**PERIOD-I (29-42 DOL)**

Gp-III (>7-10mg/kg/day) n= 32

Gp-II (>5-7mg/kg/day) n= 81

Gp-I (Standard dose Gp) n= 201

**Figure 1: Flow chart depicting recruitment of cohort initially and grouping based on daily CC-dose in two periods. (CC=caffeine citrate, Gp= Group, DOL= day of life) [note:** each participant could have more than one exclusion criterion]

**Figure 2: Mean daily weight gain of neonates in three different caffeine daily dose regimens during 15-28 days of life. (DOL= days of life)**

**Figure 3: Mean daily weight gain of neonates in three different caffeine daily dose regimens during 29-42 days of life. (DOL= days of life)**

Neonatal clinical outcomes are compared in Table A. Six neonates died in Gp-I, in each seven in group II and six in group III, which was without significant difference. In comparison with Gp-I, Gp-III had a significantly extended NICU stay. Gp-II had a longer NICU stay than Gp-I but without a significant difference. However, in comparison with Gp-I, both the other groups had significantly longer hospital stays.

|  |  |  |  |  |  |
| --- | --- | --- | --- | --- | --- |
| **Table A : Clinical outcomes in daily caffeine dose-based neonatal groups** | | | | | |
| **Dose of CC, mg/kg/day** | **Group-I**  **(≤5)** | **Group-II**  **(>5-7)** | **Group-III**  **(>7-10)** | **p-value** | |
| **N** | N=129 | N=128 | N=57 | **≤5 vs. >5-7** | **≤5 vs. >7-10** |
| **Outcome** | | | | | |
| Step down shifting | 115 (89.1%) | 114 (88.4%) | 47 (82.5%) | 0.65 | 0.073 |
| Discharged from NICU | 8 (6.2%) | 7 (5.4%) | 4 (7.0%) |  |  |
| Died | 6 (4.7%) | 7 (5.4%) | 6 (10.5%) |  |  |
| **Length of NICU stay (days)** | | | | | |
| Median (IQR) | 11 (5-29) | 12.5 (7-31.5) | 15.5 (11-36.5) | 0.12 | <0.001 |
| **Length of Hospital stay (days)** | | | | | |
| Mean ± SD | 30.2 ± 9.5 | 35.5 ± 12.5 | 41.9 ± 13.2 | <0.05 | <0.05 |
| **Discharge weight (g)** | | | | | |
| Mean ± SD | 1366.6 ± 271.3 | 1386.0 ± 362.6 | 1358.2 ± 273.4 | 0.61 | 0.77 |

Hospital stay= NICU stay + step-down unit stay; Note: Gp-I was separately compared with Gp-II and Gp-III
